# A comprehensive morphological study for basal-like breast carcinomas with comparison to nonbasal-like carcinomas

**DOI:** 10.1186/1746-1596-7-145

**Published:** 2012-10-20

**Authors:** Asli Cakir, Ipek Isik Gonul, Omer Uluoglu

**Affiliations:** 1 Department of Pathology, Gazi University School of Medicine, Besevler 06500, Ankara, Turkey

**Keywords:** Basal-like breast carcinoma, Breast, Breast carcinoma, Morphology

## Abstract

**Background:**

Breast carcinomas can be classified into five subtypes based on gene expression profiling or immunohistochemical characteristics. Among these subtypes, basal-like breast carcinomas (BLBCs) are one of the most studied group, due to their poor prognosis. The aim of this study was to investigate the prevalance, morphological and immunohistochemical features of BLBCs, in Turkish population.

**Methods:**

Five hundred invasive breast carcinomas were reviewed for several morphological features and immunostained for oestrogen and progesterone receptors, c-ERB-B2, cytokeratin5/6, cytokeratin14, vimentin and epidermal growth factor receptor (EGFR). Basal-like breast carcinoma was defined as a triple negative tumor with cytokeratin5/6 and/or EGFR positive.

**Results:**

The prevalance of BLBC was 9.6%. All medullary carcinomas and 55.6% of metaplastic carcinomas showed basal-like immunophenotype. Patients with BLBC were younger (p=0.04) and had higher-grade tumors (p<0.0001). Morphologic features associated with BLBC included increased mitosis, nuclear pleomorphism, presence of geographic and/or central necrosis, pushing margin of invasion and stromal lymphocytic response (p<0.0001). Presence of prominent nucleoli and vesicular nuclear chromatin were the cytological features correlated with basal-like phenotype (p<0.0001). On multivariate analyses, BLBCs were associated with high mitotic number (p<0.0001), the presence of vesicular chromatin (p=0.004), high tubular grade (p=0.011), lymphocytic response (p=0.031) and the absence of carcinoma insitu (p=0.039). Vimentin was positive in 53.2% of BLBCs, while cytokeratin14 was less frequently expressed (27.7%).

**Conclusions:**

BLBCs have some distinctive, but not pathognomonical, morphological features. Paying attention to these features and adding cytokeratin14 and vimentin to the immunohistochemical panel can help the definitive diagnosis of BLBCs.

**Virtual slide:**

Http://www.diagnosticpathology.diagnomx.eu/vs/5962175467857400

## Background

Breast cancer is the most frequent non-cutaneous neoplasia in women and second cause of death [1]. Breast carcinomas are heterogeneous disease such that they may have different prognoses and therapy responses despite similarities in histological types, grade and stage. Although there are 19 subtypes of breast carcinoma according to World Health Organization (WHO) 2003 classification [2], it does not entirely include the various clinical courses of this disease. Based on the distinct molecular signatures, gene expression analysis of breast carcinomas has demonstrated 5 different classes; as luminal A, luminal B, normal breast like, HER2 negative and basal-like. Recently, at 12^th^ St Gallen International Breast Cancer conference, these subtypes are reclassified as luminal A, luminal B (HER2 negative), luminal B (HER2 positive), HER2 positive (nonluminal) and triple negative (ductal) according to therapeutic options [3]. These classes usually correlate with prognosis [4,5].

The basal-like subgroup drew the special attention of researchers because of its unfavorable prognosis and limited therapy opportunities. The BLBCs constitute approximately 15% of invasive breast cancers [6]. These tumors occur frequently in premenopausal young patients [7]. They are associated with larger tumor size and distinctive histological features, including high histological grade with high mitotic rate and nuclear/cytoplasmic ratio, the presence of spindle cell or squamous metaplasia, pushing growth pattern, central acellular areas of hyalinization or necrosis and lymphocytic infiltrate [7-10]. Medullary and atypical medullary carcinomas, myoepithelial carcinomas and metaplastic carcinomas may also show the phenotype of basal-like carcinomas [8].

Basal-like breast carcinomas have variable expressions of basal cytokeratins (CKs) (CK5/6, CK14, CK17), vimentin, myoepithelial markers (smooth muscle actin, p-63), CD117, P-cadherin and/or human EGFR (HER1/EGFR) [8-13]. They are usually negative for oestrogen receptor (ER), progesterone receptor (PR) and c-ERB-B2 (so they are called triple negative, TN). Since not all TN carcinomas are basal-like, BLBC is not synonymous to TN carcinoma [14]. It has been also shown that BLBCs are associated with mutations in BRCA-1, TP53 and proteins involved in the p16/retinoblastoma pathway and met oncogene overexpression [15-18].

The gold standard of defining the BLBC is using gene expression profile (GEP) but currently using GEP is still limited due to financial concerns during daily routine; therefore immunohistochemical (IHC) markers have been used instead of the gene analyses [7,10,11]. It has been shown that a panel with four antibodies including ER, c-ERB-B2, EGFR and CK5/6 achieved to define BLBC with 55-76% sensitivity and 100% specificity [10,11]. Carey et al. updated this panel by adding PR, which is regulated by ER gene, therefore positive in most of the ER+ tumors [7]. They have defined BLBCs as ER-, PR-, c-ERB-B2-, CK5/6 and /or EGFR+ tumors [7].

To the authors’ knowledge, clinicopathological characteristics of BLBC with detailed morphological evaluation have not been studied in Turkish population before. The aim of this study is to determine the incidence of BLBCs and to define their morphological and immunohistochemical features by comparing them with the other types of breast carcinomas.

## Methods

### Case selection

Four hundred and sixty eight (468) consecutive invasive breast carcinoma patients, who underwent modified radical mastectomy, lumpectomy and excisional biopsy with or without lymph node dissection, from 2006 to 2010, were retrieved from the files of our institution. The patient’s age and gender, tumor size, surgical procedure type, the presence or absence of tumor invasion in the nipple, skin and fascia of the breast tissue were all noted from the original pathology reports. The expression of ER and PR of the tumors were determined by immunohistochemistry and c-ERB-B2 status of the tumor was determined using immunohistochemistry and/or fluorescence insitu hybridization.

The ethical review committee of Gazi University School of Medicine approved the study.

### Morphologic parameters

Two pathologist, an experienced senior pathologist and a less experienced junior pathologist reevaluated all of the tumor slides stained with hematoxylin and eosin (HE) for the following morphological features and the histological tumor type according to WHO 2003 classification [2]. The morphological features were categorized into 3 groups:

1. *Grading factors*: Histological grade was assessed using the modified Bloom-Richardson method, in which *tubule formation/grade* of the tumor, nuclear pleomorphism/atypia (*nuclear grade*), *mitotic count* were scored [19]. Mitotic count was performed on Olympus BH2 light microscope, with a graticule at x40 magnification and in 10 high-power fields (HPFs). Mitotic number was scored as 1 when it was between 0–7, 2 when between 8–14 and 3 when 15 or more.

2. *Architectural features* of the tumor:

i. *Tumor growth pattern* was assessed as *infiltrative* if there was irregular infiltration into the surrounding parenchyma or fat or *pushing* if the tumor was well circumscribed.

ii. Necrosis with its type was noted as present or absent. Large confluent areas of tumor necrosis with an irregular outline called as *geographic necrosis* and the necrosis in the middle of the tumor islands was called as *central necrosis*.

iii. *Stromal lymphocytic response* was scored as none, mild (less than 25% of the tumor), moderate (25 to 50% of the tumor) and marked (>50% of the tumor).

iv. Presence or absence of *carcinoma insitu*[20] was determined.

v. Presence of *central scar,* defined as the central fibrotic, sclerotic, predominantly acellular area of tumor, was looked for.

3. *Cytological features* of the tumor cells:

i. *Presence of nucleoli* were scored as absent or prominent if they were easily visible at low power.

ii. Amount of the *tumor cell cytoplasm* was assessed as scant, moderate or copious according to nuclear-cytoplasm ratio.

iii. Presence of *vesicular chromatin* pattern was noted.

### Tissue microarray

The specimens were routinely processed, formalin-fixed and paraffin-embedded. Invasive tumors were marked on HE stained slides for the construction of tissue microarray (Veridiam advanced tissue arrayer, VTA-100, USA). Each case was represented with 4 different 0.1 cm cores in the array blocks.

### Immunohistochemistry

Cytokeratin 5/6, CK14, EGFR and vimentin were applied on 5 μm tissue microarray sections. Sections were dewaxed in xylene substitute and hydrated with a graded series of ethanol concentrations and distillated water. Antigen retrieval was obtained in tris-EDTA (pH: 9.0) buffer for CK5/6 and citrate buffer (pH: 6.0) for EGFR, CK14 and vimentin for 20 minutes in a microwave oven. Sections were incubated with primary antibody solutions for CK5/6 (monoclonal mouse anti-human, clone D5/16 B4, Dako, Denmark), EGFR (monoclonal mouse anti-human, cloneE30, Dako), CK14 (monoclonal mouse anti-human, clone SPM 263, Spring bioscience, CA, USA) and vimentin (monoclonal mouse anti-human, cloneV9, Dako, Denmark) at a dilution of 1:100 with PBS for 1 hour at room temperature. After washing with PBS, they were incubated with secondary antibody (multispecies ultra streptavidine detection system-HRP, Zymed, Massachusetts, USA) and streptavidin-biotin complex (Zymed, Massachusetts, USA) for 20 minutes at room temperature. For immunoreaction, diaminobenzidine (diaminobenzidinetetrachloride, Zymed, Massachusetts, USA) was used as chromogen and sections were counterstained using Harris hematoxylin. Staining was performed manually. For each antibody, the intensity and percentage of staining were evaluated. Membranous staining for EGFR and cytoplasmic staining for CK5/6, CK14 and vimentin were noted. Tumors showing no staining were considered as negative.

Oestrogen receptor, PR and c-ERB-B2 results were noted from pathology reports. For ER and PR, nuclear staining more than 1% was regarded as positive. c-ERB-B2 overexpression was evaluated semiquantitatively and scores from 0 to 3 were given according to the staining intensity and the percentage of the positive tumor cells for IHC [21]. Tumors with an IHC score of 3 and/or with c-ERB-B2/CEP 17 ratio of equal or more than 2.2 in FISH analysis were regarded as positive for c-ERB-B2 amplification [21].

### Tumor classification

By using immunohistochemistry for expression profiling of BLBCs according to the criteria of Carey et al., we defined BLBC as ER, PR, c-ERB-B2 negative and CK5/6 and/or EGFR positive tumors [7]. The tumors that did not fulfill these criteria were called nonbasal-like breast carcinoma (NBBC).

### Statistical analysis

Statistical analysis was performed using the SPSS 11.5 software program. Student’s t-test and Mann–Whitney test were performed for the comparison of mean and median values, respectively. Nominal variables were compared using Pearson’s Chi-square test or Fisher’s exact test. Morphological features, which were thought to be predictive for BLBCs, were evaluated by univariate logistic regression analyses. To determine the most significant morphological features distinguishing BLBC from NBBC, multivariate logistic regression analysis with stepwise removal was used following the results obtained from univariate statistical test. For each morphological feature, the odds ratio and 95% confidence interval was calculated. Statistical significance was defined as a p-value less than 0.05 (p<0.05).

## Results

### Patients’ background

Four hundred sixty eight (468) breast carcinoma patients were retrieved. Twenty-eight (28) patients had multifocal tumors that were defined as the presence of more than one well-delineated invasive tumor foci separated by uninvolved breast tissue, regardless of the distance between foci. Thirteen (13) of 28 patients had contra lateral breast carcinoma and another 12 had 2 foci, 2 had 3 foci and 1 had 4 foci of carcinoma in the same breast. In summary, a total of 500 tumors of 468 patients were evaluated in this study.

Only 3 (0.6%) of the patients were male. Ninety-one percent (91%) of the patients had mastectomy, 7.9% had lumpectomy and 1.1% had excisional biopsy with or without axillary lymph node dissection. Patients’ demographic data and tumor characteristics were summarized in the Table 1.

**Table 1:** Demographic features and tumor characteristics of the patients with breast carcinoma

	**Total**	**BLBC**	**NBBC**	**p value**
Age	(n=468)	(n=45)	(n=423)	
	Mean:53±12 (range: 19–86) years	Mean:49.3±12.9 (range: 19–78) years	Mean:53.3±12.7 (range: 27–86) years	p=0.04
Tumor size	(n=500)	(n=47)	(n=453)	
	Mean: 2.7±1.6 cm (0.4-20)	Mean: 2.8±1.4 cm (1–8)	Mean: 2.7±1.6 cm (0.4-20)	p=0.47
	Median: 2.5 cm	Median: 2.5 cm	Median: 2.4 cm	
<2 cm	203 (40.6%)	12 (26.5%)	191 (42.2%)	p=0.07
2-5 cm	275 (55%)	33 (70.2%)	242 (53.4%)	
<5 cm	22 (4.4%)	2 (4.3%)	20 (4.4%)	
Histological grade	(n=500)	(n=47)	(n=453)	p<0.0001
1	143 (28.6%)	0 (0%)	143 (31.6%)	
2	175 (35%)	0 (0%)	175 (38.6%)	
3	182 (36.4%)	47 (100%)	135 (29.8%)	
Axillary lymph node metastasis	(n=489)	(n=44)	(n=445)	
	Mean: 5 (0–46)	Mean: 6 (0–27)	Mean: 5 (0–46)	p=0.51
0	214 (43.8%)	20 (45.5%)	194 (43.6%)	p=0.39
1-3	161 (32.9%)	16 (36.4%)	145 (32.6%)	
4-9	74 (15.1%)	3 (6.8%)	71 (16%)	
≥10	40 (8.2%)	5 (11.4%)	35 (7.8%)	
Nipple invasion	(n=456)	(n=37)	(n=419)	p=0.53
Absent	34 (7.5%)	34 (91.9%)	388 (92.6%)	
Present	422 (92.5%)	3 (8.1%)	31 (7.4%)	
Breast skin invasion	(n=500)	(n=47)	(n=453)	p=0.16
Absent	15 (3%)	44 (93.6%)	441 (97.4%)	
Present	485 (97%)	3 (6.4%)	12 (2.6%)	
Fascia invasion	(n=469)	(n=43)	(n=426)	p=0.77
Absent	39 (8.3%)	39 (90.7%)	391 (91.8%)	
Present	430 (91.7%)	4 (9.3%)	35 (8.2%)	

The most frequent histological type was invasive ductal carcinoma (IDC), which accounted for 81.6% of the tumors (408/500). Other types were invasive lobular carcinoma (23/500, 4.6%), mixed carcinoma (9 ductal+lobular, 2 ductal+mucinous, 1 mucinous+neuroendocrine, 1 tubular+cribriform) (13/500, 2.6%), mucinous carcinoma (11/500, 2.2%), metaplastic carcinoma (9/500, 1.8%), papillary carcinoma (7/500, 1.4%), medullary carcinoma (5/500, 1%), tubular carcinoma (5/500, 1%), apocrine carcinoma (4/500, 0.8%), micropapillary carcinoma (4/500, 0.8%), signet ring cell carcinoma (4/500, 0.8%), pleomorphic carcinoma (3/500, 0.6%), cribriform carcinoma (2/500, 0.4%) and neuroendocrine carcinoma (1/500, 0.2%) and atypical medullary carcinoma (1/500, 0.2%).

### Light microscopic findings

**Figure 1:**
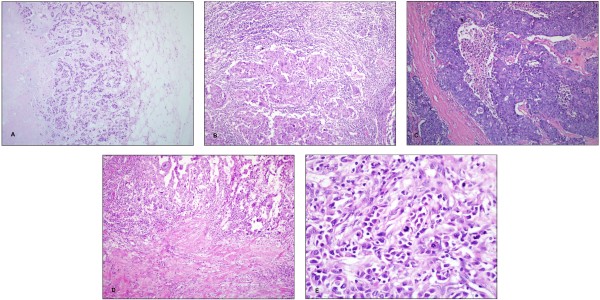
**Morphological features of BLBCs (HE staining).A**. Metaplastic carcinoma shows pushing growth and necrosis (x40). **B**. Medullary carcinoma with marked lymphocytic response and lacking tubule formation (x200). **C**. Tumor shows large central necrosis (x200). **D**. Invasive ductal carcinoma demonstrates moderate lymphocytic response and large central scar (x200). **E**. Tumor cells exhibit varying amount of cytoplasm, numerous mitosis and nuclear atypia; most of the tumor cells have vesicular chromatin and prominent nucleoli (x400).

Basal-like breast carcinoma (Figure 1).

Fortyfive (45) out of 468 patients had BLBC (9.6%) and 79.7% of TN carcinomas were basal-like. All of the patients were women. The average age for BLBCs was 49.3 ranging from 10 to 78 years.

For two patients with bilateral mastectomies, a tumor in one side was BLBC while the other in the contra lateral site was NBBC. Moreover, in a case with four separate tumors in the same breast, only two foci were BLBC. In total, 47 invasive breast tumors had basal-like immunophenotype.

The most common histological type was IDC (36/47, 76.6%). Rest of the BLBCs consisted of medullary carcinomas (5/47, 10.6%), metaplastic carcinomas (5/47, 10.6%) and pleomorphic carcinomas (1/47, 2.2%).

The average tumor size was 2.8 cm and 74.5% of the basal-like tumors were larger than 2 cm. All BLBCs showed histological grade of 3. None of the BLBCs had a histological grading score of 7 and less. The majority of tumors (44/47, 93.6%) showed solid architecture without tubule formation. A high mitotic rate was identified in most of the tumors, ranging from 8 to 60 mitoses/10 HPFs (average 25 mitoses/10 HPFs). All cases had a nuclear grade of 3, with the exception of one case (97.9%).

Most of the tumors had pushing borders (27/47, 57.5%) and 43/47 (91.5%) had some degree of stromal lymphocytic response at the tumor edge. Geographic necrosis was identified in 19/47 tumors (40.4%) whereas central necrosis was observed in 31/47 cases (66%). Majority of the BLBCs have central scar (31/47, 66%) and accompanying CIS (27/47, 57.4%).

In most of the tumors (41/47, 87.2%), cells revealed vesicular chromatin pattern. Nucleoli were prominent in 41/47 (87.2%) cases. There were slight differences between the cytoplasmic amounts of the tumor cells.

The results are summarized in Table 2.

**Table 2:** Morphologic features of the tumors and correlation with BLBCs

	**Total (n=500)**	**BLBC (n=47)**	**NBBC (n=453)**	**p value**	**Odds ratio (95% CI)**
Mitotic number	Mean: 11.5±9	Mean: 25±9	Mean: 10±8	p<0.0001	1.17 (1.12-1.21)
	Median: 9	Median: 24	Median: 8		
	Range: 1-60	Range: 8-60	Range: 1-48		
0-7	219 (43.8%)	0 (0%)	219 (48.3%)		
8-14	131 (26.2%)	3 (6.4%)	128 (28.3%)		
≥15	150 (30%)	44 (93.6%)	106 (23.4%)		
Tubule formation				p<0.0001	13.38 (4.31-44.69)
1	36 (7.2%)	0 (0%)	36 (8%)		
2	203 (40.6%)	3 (6.4%)	200 (44.1%)		
3	261 (52.2%)	44 (93.6%)	217 (47.9%)		
Nuclear grade				p<0.0001	42.80 (5.92-309.27)
1	54 (10.8%)	0 (0%)	54 (11.9%)		
2	192 (38.4%)	1 (2.1%)	191 (42.2%)		
3	254 (50.8%)	46 (97.9%)	208 (45.9%)		
Central scar				p=0.68	
Present	157 (31.4%)	16 (34%)	141 (31.1%)		
Absent	343 (68.6%)	31 (66%)	312 (68.9%)		
Central necrosis				p<0.0001	10.08 (5.24-19.38)
Present	104 (20.8%)	31 (66%)	73 (16.1%)		
Absent	396 (79.2%)	16 (34%)	380 (83.9%)		
Geographic necrosis				p<0.0001	5.23 (2.73-10.02)
Present	71 (14.2%)	19 (40.4%)	52 (11.5%)		
Absent	429 (85.8%)	28 (59.6%)	401 (88.5%)		
CIS				p<0.0001	5.29 (2.84-9.86)
Present	382 (76.2%)	20 (42.6%)	361 (79.7%)		
Absent	119 (23.8%)	27 (57.4%)	92 (20.3%)		
Stromal lymphocytic response				p<0.0001	27.02 (8.71-83.92)
None	174 (34.8%)	4 (8.5%)	170 (37.5%)		
Mild	158 (31.6%)	4 (8.5%)	154 (34%)		
Moderate	114 (22.8%)	18 (38.3%)	96 (21.2%)		
Marked	54 (10.8%)	21 (44.7%)	33 (7.3%)		
Tumor growth				p<0.0001	7.14 (3.80-13.42)
Pushing	99 (20%)	27 (57.5%)	72 (15.9%)		
Infiltrative	401 (80%)	20 (42.6%)	381 (84.1%)		
Prominent nucleol				p<0.0001	10.36 (4.31-24.91)
Present	221 (44.2%)	41 (87.2%)	180 (39.7%)		
Absent	279 (55.8%)	6 (12.8%)	273 (60.3%)		
Vesicular chromatin				p<0.0001	15.59 (6.47-37.59)
Present	179 (35.8%)	41 (87.2%)	138 (30.5%)		
Absent	321 (64.2%)	6 (12.8%)	315 (69.5%)		
Amount of cytoplasm				p=0.27	
Scant	191 (38.2%)	15 (31.9%)	176 (38.8%)		
Moderate	132 (26.4%)	15 (31.9%)	162 (35.8%)		
Copicious	177 (35.4%)	17 (36.2%)	115 (25.4%)		

### Basal-like breast carcinoma versus nonbasal-like breast carcinoma

Patients with BLBC were younger than with NBBC (p=0.04). No statistical difference was shown for the tumor size (p=0.47), the presence of lymph node metastases (p=0.51) and nipple (p=0.53), skin (p=0.16) or fascia invasion (p=0.77) between BLBCs and NBBCs.

Considering the grading factors, BLBCs had more mitotic figures (p<0.0001), more pleomorphic nuclei (p<0.0001) and more solid architecture with less tubule formation (p<0.0001) than NBBCs; in summary, BLBCs were frequently higher grade (p<0.0001).

Comparison for architectural parameters showed that BLBCs had more frequent central necrosis (p<0.0001), geographic necrosis (p<0.0001) and pushing borders (p<0.0001) than NBBCs. Accompanying CIS was rarely present in BLBCs (p<0.0001). On the other hand, moderate-severe lymphocytic response was seen more frequently in BLBCs than NBBCs (p<0.0001). There was no statistical difference for the presence of central scar between BLBC and NBBC (p=0.68).

When cytological features were compared, having prominent nucleoli and vesicular chromatin were significantly associated with BLBC (p<0.0001 for both). No difference was observed for the amount of the tumor cell cytoplasm between BLBCs and NBBCs (p=0.27).

The results are summarized in Table 2.

Univariate analyses revealed morphological features associated with BLBCs. After stepwise removal of those features not significant at 5% level, for multivariate logistic regression analysis; five features remained in the model: mitotic count, vesicular chromatin, tubular grade, lymphocytic infiltration and absence of CIS (Table 3).

**Table 3:** Multivariate analysis: Factors significantly associated with BLBCs on logistic regression analysis

	**Odds ratio**	**Wald**	**p value**	**95% CI**
Mitotic number	*1.098*	*15.633*	*<0.001*	*1.048-1.150*
Vesicular chromatin	*4.250*	*8.098*	*0.004*	*1.569-11.512*
Tubular grade	*5.361*	*6.420*	*0.011*	*1.463-19.647*
Stromal lymphocytic response	*4.177*	*4.627*	*0.031*	*1.135-15.369*
Absence of CIS	*2.344*	*4.274*	*0.039*	*1.045-5.255*

**Figure 2:**
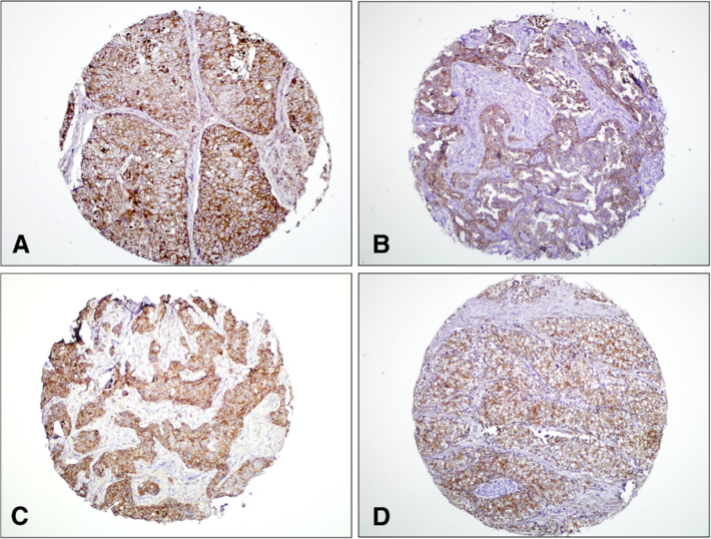
**Immunohistochemical staining of BLBCs (All photographs were taken at x100 magnification).A**. CK 5/6. **B**. EGFR. **C**. CK14. **D**. Vimentin.

Immunohistochemical findings (Figure 2).

Immunohistochemical stains for CK5/6, CK14, EGFR and vimentin were performed on 500 invasive breast carcinomas. Table 4 summarized the IHC results.

**Table 4:** Distribution of IHC markers in tumors

	**Total (n=500)**	**BLBC (n=47)**	**NBBC (n=453)**	**BLBC versus NBBC**	
				**p value**	**Odds ratio (95% CI)**
CK 5/6	257 (51%)	45 (95.7%)	212 (46.8%)		
EGFR	75 (15%)	25 (51.3%)	50 (11%)		
CK 14	48 (9.6%)	13 (27.7%)	35 (7.7%)	p<0.0001	4.56 (2.20-9.44)
Vimentin	68 (13.6%)	25 (53.2%)	43 (9.5%)	p<0.0001	10.83 (5.63-20.82)

As a definition of the BLBC in our study, all of the BLBCs showed expression of CK5/6 and/or EGFR. 48.9% of BLBCs (23/47) were positive for both CK5/6 and EGFR. 46.8% of the cases (22/47) showed CK5/6 (+)/EGFR (−) profile, however, only 4.3% of cases (2/47) were positive only for EGFR.

Cytokeratin 14 expression was identified in 27.7% (13/47) of the BLBCs and its specificity was 92.3%. Vimentin was expressed in 53.2% (25/47) of the BLBCs with a specificity of 90.5%. Both CK14 and vimentin expressions were significantly associated with the basal-like subtype (p<0.0001).

## Discussion

Basal-like breast carcinoma represents a distinctive group of invasive breast carcinomas with specific genotype and immunoprofile. It is associated with poor prognosis and currently no targeted therapy is available. There are limited numbers of studies investigating morphological features of BLBCs and to date, detailed clinicopathologic characteristics of the basal-like carcinomas have not been described in Turkish population [9,10,22,23].

Genetic, ethnic and racial factors influence breast carcinoma molecular subtypes, possibly by determining intrinsic differences in tumor biology [7]. Basal-like breast carcinomas are more frequent in African Americans (26.5%) and in African women (34%) than Non-African Americans (16.0%) [7,24]. The incidence was lower in studies from Asia, including Korea, China and Japan (14.7%, 12.6% and 8.4%, respectively) [25-27]. This study, in which we believed that the Turkish population can be reflected, only 45 out of 468 patients with invasive breast carcinomas (9.6%) exhibited basal-like immunophenotype. This incidence rate was similar to the reported incidences from the east part of the world and an earlier study from Turkey [22].

Invasive ductal carcinoma was the most frequent histological subtype identified for the BLBCs in this study (76.59%), in accordance with the literature [7,10,27,28]. Although basal-like tumors were usually shown to have worse prognosis [4,16], several studies have also revealed that carcinomas known to have a good prognosis, such as invasive lobular carcinoma, adenoid cystic carcinoma and tubular carcinomas, may also show basal-like phenotype [29-31]. Most of the medullary carcinomas with their favourable outcome, also, had basal-like phenotype with both GEP and immunohistochemistry [32-34]. The incidence of metaplastic carcinomas showing basal-like features ranges from 75 to 95% in the literature [27,35,36]. In this study, none of the invasive lobular carcinoma and tubular carcinoma cases was BLBC, but all of the medullary carcinomas (100%) and 56% of the metaplastic carcinomas had basal-like immunophenotype. A pleomorphic carcinoma was also classified as BLBC according to IHC results.

In two studies, using GEP, average age of basal-like carcinomas was detected as 46 and 59 years, respectively [7,37]. In this study, the youngest patient with BLBC was 19 and the oldest was 78 years old. We observed that BLBCs were seen in slightly younger patients compared to NBBCs (49.3 versus 53.3 years, p=0.04).

Tumor size, lymph node metastasis, local invasion are important prognostic factors for breast carcinomas. In most of the studies, it has been shown that BLBCs had larger tumor size and a tendency for visceral metastases instead of lymph node metastases [27,28,38-44]. However, contradictory observations were also present [9,37,45]. In this study, the size of BLBCs was ranged from 1 to 8 cm, with an average of 2.8 cm, with no difference from NBBCs (p=0.47). Lymph node metastasis rate for BLBCs and NBBCs were similar (54.6% versus 56.4%, respectively, p=0.51).

Identification of multifocality or bilaterality in breast carcinomas is not uncommon [46,47]. Patients with multiple tumor foci display higher incidence of lymph node positivity than unifocal tumors [46,48]. Tot et al. found that basal-like carcinomas and basal keratin positive breast tumors are often multifocal (28% and 48%, respectively) [48]. There is only one study that investigated the relationship between bilateral breast carcinomas and molecular subtypes and found that basal-like tumors are frequently discordant with their contra lateral counterparts [47]. Another study showed that contra lateral breast cancer occurred in 25% of recurrences of basal-like tumors [49]. In this study, among 468 patients, 13 had bilateral breast carcinomas, containing 11 synchronous and 2 metachronous tumors; and another 15 patients had multifocal tumor foci in the same breast. In 2 synchronous bilateral breast carcinomas, there were discordance between tumor pairs; one side had basal-like immunophenotype while the contra laterals did not. One of these NBBCs was ER and PR positive, while the other one was TN, and both of them did not express basal keratins, EGFR or vimentin. Two patients with more than one tumor foci in the ipsilateral breast had multifocal BLBC. One of these patients had 4 foci, 2 of which were BLBCs while the other two were TN carcinomas without expression of basal keratins, EGFR or vimentin. Other patient had 2 tumor foci and both were BLBCs. Despite of same hormonal and environmental influences on the ipsilateral and/or contralateral breast, this heterogeneity may support a stem cell hypothesis on carcinogenesis in which continuing mutations may result in development of different types of carcinomas. We believe that studies in which stem cell surface markers are being investigated should be constructed to highlight this issue.

The typical histological appearance of BLBCs has been reported to be a circumscribed, solid lesion with pushing border and a large central ‘geographic’ necrosis or sclerosis. They have nuclear pleomorphism with high histological grade and high mitotic rate (usually more than 40–45 mitosis per 10 HPFs), consistent with their aggressive behavior [7,9,10,27,28,37,50]. Other common morphological features are stromal lymphocytic response, tumor cells with high nucleus to cytoplasmic ratio, vesicular chromatin and prominent nucleoli [9,10,23,42,45].

All basal-like tumors, we have analyzed in this study, were grade 3 with a specificity of 70.2%. The mitotic rate was also prominently increased, 93% of basal-like tumors had 15 mitoses and more per 10 HPFs (ranging from 8 to 60 mitoses/10 HPFs). Most of the tumor cells were generally disposed in nests, ribbon like and solid structures with a tubular score of 3 (93.6%). All except one (97.9%), BLBCs had a nuclear grade of 3. All of the grading factors were statistically determinant for BLBC (p<0.0001), supporting that BLBCs are solid, high-grade tumors with high mitotic count and pleomorphic-atypical nuclei. In this study, both geographic and central necroses were more common in BLBC than NBBC (respectively, 40.4% vs. 14.5% and 66% vs. 16.1%, p<0.0001). We observed that large, central acellular zones can be found in BLBCs, in contrast to the findings of Livasy et al. [10], although it was not a discriminator morphology for them (p=0.68). Consistent with the literature [9,10,23], any degree of stromal lymphocytic response was the most common morphological feature for BLBCs among others investigated in this study (91.5%). Similarly, patients with basal-like carcinoma had increased tendency to grow with pushing borders than NBBC (57.5% versus 15.9% respectively, p<0.0001). Although CIS is regarded as a precursor lesion for most of the invasive breast carcinomas [2], the reported incidence of accompanying CIS in BLBCs is low [23,48,51]. Compatible with these data, CIS did not accompany to 57.4% of the BLBCs in this study, supporting the idea that BLBC transforms to invasive cancer in an early period, without remaining in pre-invasive CIS stage. The amount of the cytoplasm of the tumor cells did not show a meaningful difference between BLBCs and NBBCs in this study (p=0.27). As a summary, BLBCs exhibits these main histological characteristics: high histologic and nuclear grade, lack of tubule formation, frequent mitotic figures, central and geographic necrosis, pushing tumor borders and lymphocytic infiltrate. Cytologically, they have prominent nucleoli and nuclear vesicular chromatin. Carcinoma in situ usually does not accompany to the tumor. Among these features, on multivariate analyses, the most important factors were mitotic number (OR: 1.098, p<0.0001), vesicular chromatin (OR: 4.250, p=0.004), tubule grade (OR: 5.361, p=0.011), stromal lymphocytic response (OR: 4.177, p=0.031) and absence of CIS (OR: 2.344, p=0.039). Fulford et al. also performed multivariate analyses and reported that presence of squamous metaplasia, central scar, tumor necrosis, high mitotic count and absence of prominent cytoplasm are strongly associated with BLBC [9].

Although most of the BLBCs are TN, approximately 71-85% of TN carcinomas have basal-like phenotype [11,52-54]. In this study, BLBCs constituted 79.7% of TN carcinomas and this proportion was in the reported range from previous studies.

Immunohistochemical markers, such as mammoglobulin, ER, PR and CK7, can be used for the detection of breast origin of a tumor [55]. Some other markers, such as CK5/6 and CK14, have been shown to be independently associated with poor outcome when expressed in breast carcinomas. In non-neoplastic breast ducts, these two markers are also expressed in the basal layer, so called basal CKs [56-59]. In this study, 27.6% of the BLBCs were immunoreactive to CK14 and 95.7% were to CK5/6. It should be noted that CK5/6 and CK14 expressions were also identified 46.8% and 7.7% of NBBCs. Restricted expression of CK14 to mature myoepithelial cells and the broader expression of CK5/6 in bi-potential progenitor cells may explain the difference in basal keratin expression in the tumors [60]. However, the frequency of CK5/6 expression in our study was much higher than the reported series in the literature [28,56,57]. On the other hand, it has been known that basal CKs are not expressed in all basal-like tumors classified by gene microarray analysis [10]. Therefore, we actually need additional IHC markers to identify basal-like tumors. Epidermal growth factor receptor (EGFR) is a member of the c-ERB-B family of tyrosine kinase receptor proteins, and has a role in tumor cell survival and proliferation [61]. An association between EGFR expression and the basal like phenotype has been demonstrated in several studies [10,11]. The expression rates for EGFR ranged from 5% to 65% in breast carcinomas and from 45% to 72% in BLBCs, depending on the methodology used in different studies [10,11,27,62-64]. Shao et al. also showed that immunohistochemical expression of EGFR correlated with and predicted EGFR amplification [65]. In this study, 51.3% of BLBCs were EGFR positive, whereas only 11% of NBBC cases expressed EGFR. This high percentage of EGFR expression in BLBCs is important, not only for the diagnosis of BLBCs, but also in the treatment of this high grade and TN cancers [65]. Lv et al. showed that EGFR gene amplification is more frequent than EGFR gene mutations [64]. We believe that potentially promising anti-EGFR therapies should be introduced for the cases in which gene copy numbers were examined by molecular studies for now, similar to c-ERB-B2.

Vimentin expression in breast carcinomas may have an association with poor prognosis, hormone receptor negativity and co-expression of EGFR, which are consistent features for basal-like tumors [66,67]. In several studies, more than 90% of the basal-like carcinomas were found to have diffuse and strong vimentin expression [10,68]. In our series, 53.2% of BLBCs were immunoreactive to vimentin, while 9.5% of NBBCs also showed positivity (p<0.0001). In accordance with the basal CKs, myoepithelial or stem cell origin and epithelial to mesenchymal transition can explain the vimentin expression in basal-like tumors [10]. In this study, vimentin had a concordance of 54.2% with CK5/6 and 79.4% with EGFR (p<0.0001) for BLBCs. If vimentin or CK14 were included in the IHC panel for identifying basal-like tumors, three more TN cases would be considered as BLBCs. Moreover, these high-grade tumors had similar architectural and/or cytological features to BLBCs. Therefore; we suggested that vimentin and CK14 could be added to the diagnostic panel of antibodies to increase the diagnostic accuracy of basal-like tumors. Moreover, using integrating digital image analysis, while evaluating multiple IHC markers, can help to determine subgroups of breast carcinomas more accurate [69].

## Conclusion

Basal-like breast carcinomas appear to occur less frequently in Turkish women, which could reflect intrinsic differences in tumor biology related to racial/ethnic factors. Synchronously, different molecular subtypes of breast carcinomas can occur in bilateral and/or multifocal breast carcinomas. Light microscopic findings of the study suggest that there are many significant morphological differences between basal-like and nonbasal-like breast carcinomas. In addition to these features, vimentin and CK14 may be used as additional markers to include in the IHC panel for distinguishing BLBCs.

## Abbreviations

BLBC: Basal-like breast carcinoma; EGFR: Epidermal growth factor receptor; WHO: World Health Organization; CK: Cytokeratin; ER: Oestrogen receptor; PR: Progesterone receptor; TN: Triple negative; GEP: Gene expression profiling; IHC: Immunohistochemical; HE: Hematoxylin and eosin; HPF: High power field; CIS: Carcinoma insitu; IDC: Invasive ductal carcinoma; NBBC: Non basal-like breast carcinoma.

## Competing interests

The authors declare that they have no competing interests.

## Authors’ contributions

AC designed and performed the research, analyzed the data and wrote the paper. IIG contributes in writing the paper. OU analyzed the data and designed the research. All authors read and approved the final manuscript.
